# Genome Sequence of the Acetogenic Bacterium *Moorella mulderi* DSM 14980^T^

**DOI:** 10.1128/genomeA.00444-16

**Published:** 2016-05-26

**Authors:** Genis Andrés Castillo Villamizar, Anja Poehlein

**Affiliations:** Genomic and Applied Microbiology & Göttingen Genomics Laboratory, Georg-August University Göttingen, Göttingen, Germany

## Abstract

Here, we report the draft genome sequence of *Moorella mulderi* DSM 14980^T^, a thermophilic acetogenic bacterium, which is able to grow autotrophically on H_2_ plus CO_2_ using the Wood-Ljungdahl pathway. The genome consists of a circular chromosome (2.99 Mb).

## GENOME ANNOUNCEMENT

The reduction of CO_2_ mediated by acetogenic microorganisms is gaining more interest as a valuable tool for the generation of renewable energy and value-added chemicals (1–3). Thus, homoacetogenic bacteria that use the Wood-Ljungdahl pathway for the CO_2_ fixation process have proven to be a main component in this research field (3–8). Among the numerous species of homoacetogens, three organisms have been relatively well studied (*Moorella thermoacetica*, *Acetobacterium woodii*, and *Clostridium ljungdahlii*) (9–13). However, several relevant species remain poorly studied, and the genetic information of many of them remains almost nonexistent or is very limited. Therefore, in this study, we report the draft genome sequence of *Moorella mulderi* DSM 14980^T^ a thermophilic homoacetogenic anaerobic bacterium originally isolated from a bioreactor with methanol as the energy source (14). Similar to *M. thermoacetica*, *M. mulderi* DSM 14980^T^ is able to grow on several substrates, including methanol, H_2_-CO_2_, pyruvate, and glucose. However, several differences have been reported. The optimal temperature of *M. mulderi* DSM 14980^T^ (65°C) is higher than the optimal temperature reported for *M. thermoacetica* (55 to 60°C). Moreover, in contrast to *M. thermoacetica, M. mulderi* DSM 14980^T^ is able to grow on lactate but cannot use nitrate as an electron acceptor (14).

The MasterPure complete DNA purification kit (Epicentre, Madison, WI, USA) was used to isolate the chromosomal DNA of *M. mulderi* DSM 14980^T^. Isolated DNA was used to generate Illumina shotgun sequencing libraries. Sequencing was performed by employing a MiSeq system using MiSeq reagent kit version 3 (600 cycles), as recommended by the manufacturer (Illumina, San Diego, CA, USA), resulting in 2,785,408 paired-end reads (300 bp) that were trimmed using Trimmomatic 0.32 (15). *De novo* assembly performed with the SPAdes genome assembler software version 3.6.2 (16) resulted in 72 contigs (>500 bp) and an average coverage of 188.5-fold.

The genome of *M. mulderi* DSM 14980^T^ probably consists of a circular chromosome of (2.99 Mb) with an overall G+C content of 53.32%. Gene prediction and annotation were performed using Rapid Prokaryotic Genome Annotation (Prokka) (17). The genome harbored 3 rRNA genes, 52 tRNA genes, 2,240 protein-coding genes with predicted functions, and 859 genes coding for hypothetical proteins. The cluster of genes encoding enzymes of the methyl and carbonyl branches of the Wood-Ljungdahl pathway is conserved within acetogenic bacteria (18). Therefore, *M. mulderi* DSM 14980^T^ shows an arrangement identical to the pattern previously identified in *M. thermoacetica* strains ATCC 39073 and DSM 521^T^ (10, 18). The cluster is composed of eight genes (*acsFABCV*, *cooC*, and *acsDE*) encoding the subunits of the CO dehydrogenase–acetyl-coenzyme A (CoA) synthase complex. The genes encoding the two subunits of the methylene-THF reductase (*metVF*) are located four genes downstream of this cluster.

The genome analysis revealed that *M. mulderi* DSM 14980^T^ has a bigger genome size than *M. thermoacetica* DSM 521^T^ (2.52 Mb) and *M. thermoacetica* DSM 2955^T^ (2.62 Mb) (10, 19).

### Nucleotide sequence accession numbers.

This whole-genome shotgun project has been deposited at DDBJ/ENA/GenBank under the accession no. LTBC00000000. The version described in this paper is version LTBC01000000.
